# EZH2/EHMT2 Histone Methyltransferases Inhibit the Transcription of DLX5 and Promote the Transformation of Myelodysplastic Syndrome to Acute Myeloid Leukemia

**DOI:** 10.3389/fcell.2021.619795

**Published:** 2021-08-02

**Authors:** Zhuanzhen Zheng, Ling Li, Guoxia Li, Yaofang Zhang, Chunxia Dong, Fanggang Ren, Wenliang Chen, Yanping Ma

**Affiliations:** Department of Hemapathotology, Second Hospital of Shanxi Medical University, Taiyuan, China

**Keywords:** myelodysplastic syndromes, acute myeloid leukemia, EZH2, EHMT2, DLX5, H3K27me3, H3K9me2

## Abstract

Myelodysplastic syndrome (MDS) is characterized by clonal hematopoiesis and impaired differentiation, and may develop to acute myeloid leukemia (AML). We explored the mechanism of histone methyltransferase EZH2/EHMT2 during the transformation of MDS into AML. Expression of EZH2/EHMT2 in patients and NHD13 mice was detected. EZH2 and EHMT2 were silenced or overexpressed in SKM-1 cells. The cell proliferation and cycle were evaluated. Levels of DLX5, H3K27me3, and H3K9me2 in SKM-1 cells were detected. Binding of DLX5 promoter region to H3K27me3 and H3K9me2 was examined. Levels of H3K27me3/H3K9me2 were decreased by EZH2/EHMT2 inhibitor (EPZ-6438/BIX-01294), and changes of DLX5 expression and cell proliferation were observed. EZH2 was poorly expressed in MDS patients but highly expressed in MDS-AML patients. EHMT2 was promoted in both MDS and MDS-AML patients. EZH2 expression was reduced and EHMT2 expression was promoted in NHD13 mice. NHD13 mice with overexpressing EZH2 or EHMT2 transformed into AML more quickly. Intervention of EZH2 or EHMT2 inhibited SKM-1 cell proliferation and promoted DLX5 expression. When silencing EZH1 and EZH2 in SKM-1 cells, the H3K27me3 level was decreased. EZH2 silencing repressed the proliferation of SKM-1 cells. Transcription level of DLX5 in SKM-1 cells was inhibited by H3K27me3 and H3K9me2. Enhanced DLX5 repressed SKM-1 cell proliferation. In conclusion, EZH2/EHMT2 catalyzed H3K27me3/H3K9me2 to inhibit the transcription of DLX5, thus promoting the transformation from MDS to AML.

## Introduction

Myelodysplastic syndrome (MDS) comprises a group of heterogeneous myeloid neoplasms sharing the common characteristics of bone marrow failure, including hematopoietic dysfunction, morphologic dysplasia, and peripheral blood cell reduction (Ogawa, 2019). MDS patients with multiple lineage cytopenias, high percentage of bone marrow blasts or characteristic chromosomal abnormalities, usually develop rapidly into acute myeloid leukemia (AML) and eventually die of the disease in the absence of bone marrow transplantation (Griffiths and Gore, 2013). AML is a completely malignant and aggressive blood cancer, featured by the extensive accumulation of developmentally arrested and immature blasts in bone marrow (Platzbecker et al., 2017). It has demonstrated that more than half of MDS and AML cases are the elderly, and the prevalence of MDS and AML may continue increasing due to the global population aging (Kubasch and Platzbecker, 2018). Identifying the potential molecular events associated with MDS/AML progression can better understand the pathogenesis of this disease and improve the therapeutic effect.

NHD13 mouse is an animal model of MDS based on NUP98-Hoxd13 fusion gene, which has the key characteristics of MDS and may have leukemia transformation within 14 months, and consequently it is often used in medical research (Lin et al., 2005; Slape et al., 2008). The aberrant expression of EZH2 is closely related to the transformation from MDS to AML (de Souza Fernandez et al., 2019). EZH2 is a histone methyltransferase, which can tri-methylate histone H3 of lysine 27 (H3K27me3) and silence target genes related to various functions including cell cycle, proliferation, and differentiation (Tremblay-LeMay et al., 2018). Sashida et al. (2014) have shown that EZH2 deletion accelerates the development of MDS, but weakens the tendency of MDS to AML. After EZH2 knockout, the proportion of NHD13 mice transformed into AML is decreased (Ling et al., 2019). Therefore, the overexpression of EZH2 may be a potential biomarker for the transformation of MDS to AML.

EHMT2, also known as G9a, is a histone methyltransferase that catalyzes the methylation of histone 3 lysine 9 (H3K9) (Cao et al., 2019). EHMT2 exerts methyltransferase activity in AML cells and promotes the transcription of leukemia related genes (Lehnertz et al., 2014). Importantly, a recent literature has exhibited that EHMT2 and EZH2 interact physically and share targets for epigenetic silencing (Mozzetta et al., 2014). The dual inhibition of EZH2 and EHMT2 can induce gene transcription and inhibit tumor cell growth more effectively (Curry et al., 2015).

The hypermethylation of DLX5 promoter is related to its low expression and represents a common event in AML and MDS, which also contributes to the transformation of MDS into leukemia (Zhang et al., 2020). A study on lung cancer has shown that lysine demethylase 4A (KDM4A) increases the transcriptional activity of DLX5 by promoting the demethylation of DLX5 (Sun et al., 2020). Accordingly, we speculated that DLX5 may also be regulated by histone methyltransferase in the transformation from MDS to leukemia. At present, the specific mechanism of EZH2 in the transformation of MDS to AML remains unclear. Whether EZH2 and EHMT2 play an epigenetic regulatory role synergistically is worth further exploring. Therefore, this study explored the role of EZH2/EHMT2 in the transformation of MDS to AML, which shall provide a theoretical basis for the management of MDS and MDS-AML.

## Materials and Methods

### Ethics Statement

The study got the approval of the Clinical Ethical Committee of the Second Hospital of Shanxi Medical University. Informed consent was signed by each eligible participant.

### Tissue Samples

Thirty-three bone marrow samples were collected from the Second Hospital of Shanxi Medical University from 2015 to 2018. All the samples were confirmed as MDS (*N* = 11), MDS-AML (*N* = 11), or cancer-free individuals (*N* = 11) by bone marrow puncture and/or biopsy. Among them, MDS-AML referred to the patients who were definitely diagnosed with MDS and then turned into AML. MDS patients and MDS-AML patients were matched according to age and gender. Bone marrow samples were extracted with human peripheral blood lymphocyte separation solution (TBD, Tianjin, China) by density gradient method. Total RNA was isolated from monocytes using RNAiso Plus reagent (Takara, Dalian, China) and reverse transcribed into cDNA using a reverse transcription quantitative polymerase chain reaction kit (Takara), and then stored at −80°C.

### Experimental Animals

NHD13 mice were purchased from Jackson Laboratory and C57BL/6 mice were purchased form Kunming Institute of Zoology, Chinese Academy of Sciences [SYXK (Yunnan) K2015-0003]. Mice were raised in a specific pathogen-free animal facility. Food and water were provided *ad libitum*. The expressions of EZH2 and EHMT2 in peripheral blood of mice were detected at 4 months old. Then peripheral blood was collected regularly and the blood condition was detected. Blood samples were collected from mice at the age of 14 months (420 days), and then all the mice were euthanized by an intraperitoneal injection of pentobarbital (800 mg/kg) (Kopaladze, 2000; Zatroch et al., 2017).

### Cell Culture

Human MDS/AML cells (SKM-1 cells) were purchased from BeNa Culture Collection (Beijing, China) and cultured in Roswell Park Memorial Institute-1640 medium containing 10% fetal bovine serum in 95% humidified air with 5% CO_2_ at 37°C.

The small interfering RNA (siRNA)-NC-1, si-EZH1, si-NC-2, si-EZH2, si-NC-T2, and si-EHMT2 were designed and synthesized by GenePharma (Shanghai, China), and the sequence is shown in Supplementary Table 1. Overexpression vectors of EZH2 (pcDNA3.1-EZH2) and EHMT2 (pcDNA3.1-EHMT2) and empty vectors were constructed by Zoman Biotechnology Co., Ltd. (Beijing, China). Then, the constructed vectors and siRNAs were transfected into cells using Lipofectamine 2000 (Invitrogen Inc., Carlsbad, CA, United States).

SKM-1 cells were treated with EZH2 inhibitor EPZ-6438 (5 μM, Yeasen Biotech Co., Ltd., Shanghai, China) and EHMT2 inhibitor BIX-01294 (2.5 μM, Sigma-Aldrich, Merck KGaA, Darmstadt, Germany). Briefly, cells in logarithmic growth phase were seeded into 96-well plates (1 × 10^5^ cells/mL) supplemented with culture medium, and treated with 5 μM EPZ-6438 for 4 days (Knutson et al., 2014) and 2.5 μM BIX-01294 for 3 days, respectively (Huang et al., 2017). The treated cells were allocated as EPZ group and BIX group, respectively.

### Cell Counting Kit-8 (CCK-8) Assay

The treated cells were seeded into the 96-well plates (2 × 10^3^ cells/well), and CCK-8 solution was added at 24, 48, and 72 h. The optical density of each well was measured at 450 nm. The experiment was repeated three times in each group.

### Flow Cytometry

SKM-1 cells under different treatments were fixed with 70% ethanol, stained with 300 mL propidium iodide (MultiSciences Biotech Co., Ltd., Hangzhou, Zhejiang, China) in the dark, and detected on the flow cytometer (MoFlo Astrios EQ, Beckman Coulter, Inc., CA, United States) to analyze the cell cycle.

### 5-Ethynyl-2′-Deoxyuridine (EdU) Labeling Assay

The culture medium of SKM-1 cells under different treatments was removed, and the cells were washed with phosphate-buffered saline (PBS), incubated with EdU solution for 2 h, and then photographed under a fluorescence microscope (Olympus, Tokyo, Japan).

### Colony Formation Assay

SKM-1 cells under different treatments were seeded into the 12-well plates (1 × 10^4^ cells/well), and incubated at 37°C for 1 week until cell colonies were observed. The colonies were stained with crystal violet and counted.

### Chromatin Immunoprecipitation (ChIP)

SKM-1 cells were subjected to ChIP assay referring to previous literature (Dagdemir et al., 2013). Cells were detached with trypsin and counted using Millipore Scepter 2.0 Cell (Thermo Fisher Scientific, Jiangsu, China). And 1 × 10^6^ cells were used for each treatment. Cells were incubated for 8 min in the medium, fixed with formaldehyde and then crosslinked with 1.25 M glycine for 5 min at room temperature. All chromatin preparation and ChIP reaction were carried out at 4°C. The crosslinked cells were washed with PBS-inhibitor (NaBu 20 mM), and the cell membrane was cleaved with the HighCell ChIP kit. Chromatin was prepared in TPX tube with shear buffer S1 and 1 × protease inhibitor, and then broken into fragments of about 500 bp by ultrasound. The size of the fragments was examined on agarose gel, and the cut chromatin was frozen at −80°C. ChIP reaction was performed using the Diagenode kit on the SX-8X IP STAR compact automation system (Diagenode) for all IP procedures. According to the HighCell ChIP kit protocol, IP DNA was purified using DNA Isolation buffer with 2 μg antibody [anti-H3K27me3 (10 μg for 25 μg of chromatin, ab6002, Abcam, Cambridge, MA, United States) or anti-H3K9me1 (5 μg for 10^6^ cells, ab9045, Abcam) or anti-H3K9me2 (4 μg for 25 μg of chromatin, ab1220, Abcam)] and non-immune immunoglobulin G (IgG). Each auto-ChIP sample was performed using the Auto Histone ChIP-seq kit and contained 1 μg input chromatin. The reaction lasted for 2 h. The antibody was coated with protein A-coated magnetic beads, and then incubated for 10 h at 4°C for IP reaction. Afterward, 25 μL system of DNA IP or DNA input (total DNA), 1 × SYBR Green Supermix (Applied Biosystems, Inc., Carlsbad, CA, United States) and TSH2B (pp-1041–500, Diagenode; positive control of methylation) promoter were used for reverse transcription quantitative polymerase chain reaction (RT-qPCR), with the sequence of DLX5 promoter region as primer.

### RT-qPCR

Total RNA was extracted from cells using TRIzol one-step reagent (Invitrogen), and then the concentration and purity of RNA were determined using UV analysis and formaldehyde deformation electrophoresis. The fluorescent qPCR reaction was performed on the instructions of the RT-qPCR kit (Thermo Fisher scientific). Primers (Table 1) were designed and synthesized by Sangon Biotech (Shanghai, China). Amplification curve and dissolution curve were confirmed after reaction. The relative expression of genes was calculated by 2−ΔΔ*Ct* method, with glyceraldehyde-3-phosphate dehydrogenase (GAPDH) as the internal reference.

**TABLE 1:** Primer sequence for RT-qPCR.

Gene	Primer sequence
GAPDH	F: 5′-GGGAGCCAAAAGGGTCAT-3′ R: 5′-GAGTCCTTCCACGATACCAA-3′ F: 5′-ATGGGCCAGACTGGGAAGAAA-3′
EZH2	R: 5′-GGAGGTAGCAGATGTCAAGGG3′ F: 5′-ATGGAGGATTACAGCAAGATGG-3′
EZH1	R: 5′-GGGGCCTGGGAGGGCTAAAGGA-3′ F: 5′-ATGCGGGGTCTACCGAGAGGG-3′
EHMT2	R: 5′-AGAGAGGGTGTGGTCCGTTCTC-3′
DLX5	F: 5′-ATGACAGGAGTGTTTGACAGAAG-3′ R: 5′-CTAATAGAGTGTCCCGGAGGCCA-3′
DLX5 ChIP primer 1	F: 5′-TTCTACACTCGCCTTTGGTG-3′ R: 5′-CAGCACAAGGCTCTGTGATG-3′
DLX5 ChIP primer 2	F: 5′-CCCACTCCACAACAAGCAA-3′ R: 5′-GCACAGCCTTGGTTAAATCC-3′

### Western Blot Analysis

Cells in each group were lysed in radio-immunoprecipitation assay buffer containing protease inhibitors (Sigma-Aldrich). The lysate was centrifuged at 16000 *g* and 4°C for 20 min to collect the supernatants. The concentration of protein extracted from cells was tested using the Pierce bicinchoninic acid assay kit (Beyotime, Shanghai, China). Then, the protein was separated using sodium dodecyl sulfate-polyacrylamide gel electrophoresis and transferred onto polyvinylidene difluoride membranes (Millipore, Bedford, MA, United States). The membranes were blocked with 5% skim milk for 2 h and cultured with the primary antibodies at 4°C overnight. Thereafter, the membranes were cultured with the secondary antibody for 1 h, and developed and visualized using the enhanced chemiluminescence reagent. The gray value of the target band was analyzed by Image J software (National Institutes of Health, MD, United States). The antibodies used were as follows: H3K27me3 (1:1000, ab6002, Abcam), β-actin (1:1000, ab8227, Abcam), H3K9me1 (1:1000, ab9045, Abcam), H3K9me2 (1:1000, ab1220, Abcam), EZH1 (1:1000, ab189833, Abcam), EZH2 (1: 1000, ab150433, Abcam), and EHMT2 (1:1000, ab185050, Abcam).

### Statistical Analysis

SPSS 21.0 (IBM Corp., Armonk, NY, United States) was utilized for data analysis. Shapiro–Wilk test showed that the data in each group were in normal distribution. Data are expressed as mean ± standard deviation. The Mann–Whitney *U* test or *t* test was adopted for analysis of comparisons between two groups. The one-way analysis of variance (ANOVA) was applied for comparisons among multi-groups, followed by Tukey’s multiple comparisons test. Kaplan–Meier curve was used to analyze the AML transformation and survival of mice, and Log-rank method was used to test the differences between groups. The *p* value was obtained from a two-tailed test, and *p* < 0.05 meant a statistical difference.

## Results

### EZH2 and EHMT2 Were Upregulated in MDS-AML Patients

**FIGURE 1:**
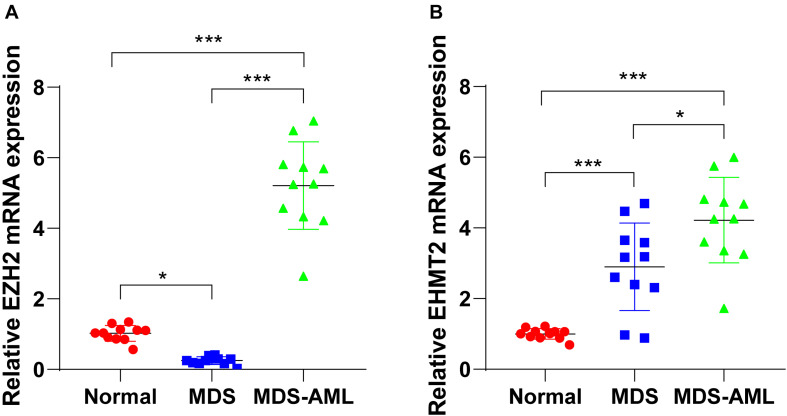
EZH2 and EHMT2 were upregulated in MDS patients and MDS-AML patients. **(A,B)** Expressions of EZH2 and EHMT2 in bone marrow of MDS patients, MDS-AML patients, and non-cancer individuals were detected using RT-qPCR. Each experiment was repeated three times. Data are analyzed using one-way ANOVA, followed by Tukey’s multiple comparisons test, *p < 0.05, ***p < 0.001.

The detection of bone marrow samples collected from MDS and MDS-AML patients in the Second Hospital of Shanxi Medical University from 2015 to 2018 showed that EZH2 expression in bone marrow of MDS patients was notably lower than that of healthy non-cancer individuals (*p* < 0.05; Figure 1A), but EZH2 expression in MDS-AML patients showed a trend of high expression (*p* < 0.001; Figure 1B). EHMT2 was always highly expressed in MDS and MDS-AML patients (both *p* < 0.001; Figures 1A,B). The general information of participants is shown in Table 2.

**TABLE 2:** General information of the cases.

	Normal (*n* = 11)	MDS (*n* = 11)	MDS-AML (*n* = 11)	*p*
Age	50.5 ± 10.5	47.4 ± 12.4	51.8 ± 13.8	0.693
Sex (Male/Female)	6/5	7/4	7/4	0.881
BMI (kg/m^2^)	22.35 ± 2.01	23.16 ± 1.97	23.51 ± 1.59	0.340

### NHD13 Mice With High Expressions of EZH2 and EHMT2 Transformed Into AML More Quickly

**FIGURE 2:**
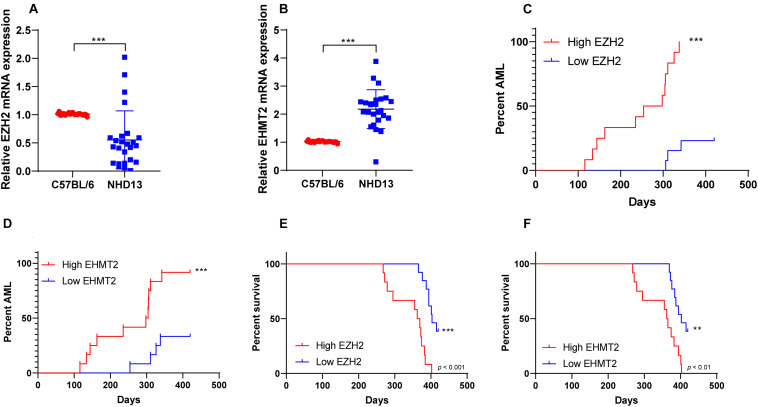
NHD13 mice with high expression of EZH2 transformed into AML more quickly. The survival of NHD13 mice (*N* = 25) was observed within 420 days, and the expressions of EZH2 and EHMT2 in peripheral blood of mice were detected at the age of 4 months. NHD13 mice were allocated into high/low expression groups according to the median expressions of EZH2 and EHMT2. **(A,B)** The expressions of EZH2 and EHMT2 in peripheral blood of mice (at the age of 4 months) were detected using RT-qPCR; **(C,D)** The number and time of transforming from MDS into AML of NHD13 mice in different groups within 14 months were recorded; **(E,F)** Survival curves were used to analyze the survival rates of NHD13 mice. Data in panels **(A,B)** are analyzed using Mann–Whitney *U* test or *t* test, and data in panels **(C–F)** were analyzed using Log-rank test, **p < 0.01, ***p < 0.001.

NHD13 mice faithfully reproduced all the key features of MDS, including decreased peripheral blood cells, abnormal bone marrow hyperplasia and increased apoptosis, and conversion to acute leukemia at 4–14 months of age (Lin et al., 2005; Slape et al., 2008). The survival of NHD13 mice (*N* = 25) were observed within 420 days, and the expressions of EZH2 and EHMT2 in peripheral blood of mice were detected at the age of 4 months. The results showed that EZH2 expression in NHD13 mice was lower than that in healthy C57BL/6 mice (*p* < 0.05; Figure 2A). According to the median expression of EZH2 in NHD13 mice, the mice with the expression of EZH2 higher than the median were allocated as the EZH2 high expression group (relative EZH2 mRNA expression >0.45, *N* = 12), and the mice with the expression of EZH2 lower than the median were allocated as the EZH2 low expression group (relative EZH2 mRNA expression ≤0.45, *N* = 13). Comparing the two groups of mice, it was found that NHD13 mice with high expression of EZH2 transformed from MDS to AML in a short period of time, while the mice with low expression of EZH2 took a relatively long time to transform into AML, even without the presence of AML transformation (Figure 2C). Additionally, the survival rate of NHD13 mice with high expression of EZH2 was significantly lower (*p* < 0.05; Figure 2E). EHMT2 expression in NHD13 mice was notably increased compared with that in healthy mice (*p* < 0.05; Figure 2B). Similarly, according to the median expression of EHMT2, mice were designated into EHMT2 high expression group (relative EHMT2 mRNA expression >2.13, *N* = 12) and EHMT2 low expression group (relative EHMT2 mRNA expression ≤2.13, *N* = 13). It was found that mice with high expression of EHMT2 developed from MDS to AML more quickly (Figure 2D), and the survival rate of NHD13 mice with high expression of EHMT2 was significantly reduced (*p* < 0.05; Figure 2F). These results suggested that the expressions of EZH2 and EHMT2 were related to the transformation from MDS to AML.

### Interference of EZH2 Expression Inhibited SKM-1 Cell Proliferation

**FIGURE 3:**
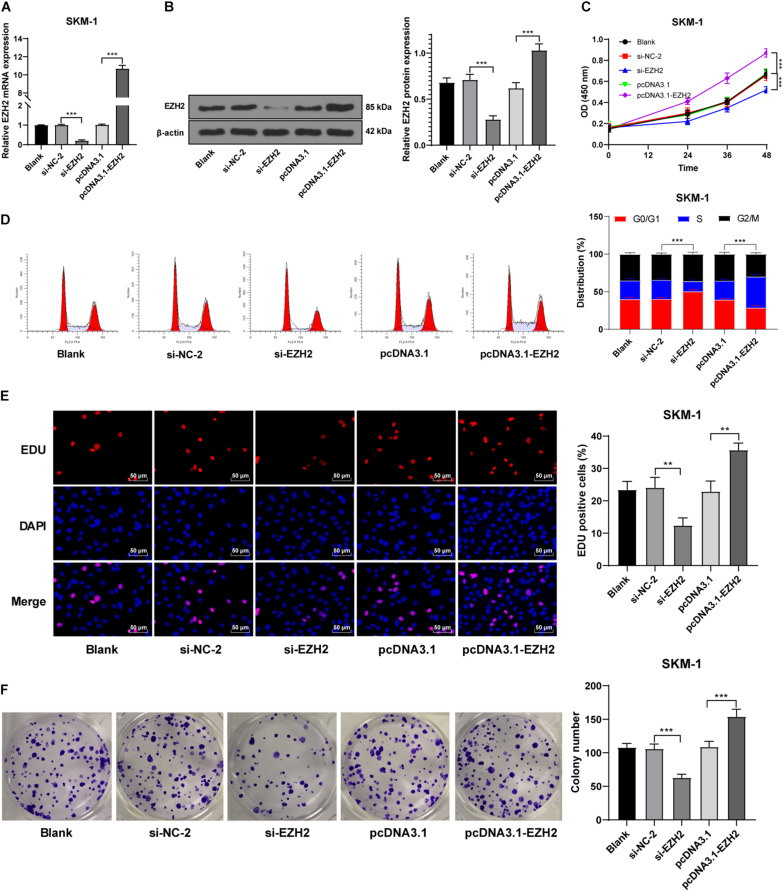
Silencing EZH2 expression inhibited SKM-1 cell proliferation. **(A,B)** Transfection efficiency of si-EZH2 or overexpression of EZH2 was confirmed using RT-qPCR and Western blot; **(C)** Viability of SKM-1 cells under different treatments was measured using CCK-8 assay; **(D)** Cell cycle of SKM-1 cells was detected using flow cytometry; **(E,F)** Proliferation ability of SKM-1 cells was measured using EdU and colony formation assay. The cell experiments were repeated three times. Data are expressed as mean ± standard deviation. Data were analyzed using one-way ANOVA, followed by Tukey’s multiple comparisons test, **p < 0.01, ***p < 0.001.

Our data showed that EZH2 had an opposite trend in MDS and AML. To further investigate the role of EZH2 in MDS and MDS-AML, we transfected SKM-1 cells with si-EZH2 or pcDNA3.1-EZH2. The results of RT-qPCR and Western blot confirmed the transfection efficiency (*p* < 0.05; Figures 3A,B). SKM-1 cells transfected with si-EZH2 showed significantly reduced proliferation ability and blocked cell cycle, while SKM-1 cells transfected with pcDNA3.1-EZH2 had the opposite trend (all *p* < 0.05; Figures 3C–F). It was indicated that the deletion of EZH2 inhibited MDS cell proliferation, while the overexpression of EZH2 facilitated cell proliferation.

### Interference of EHMT2 Expression Inhibited SKM-1 Cell Proliferation

**FIGURE 4:**
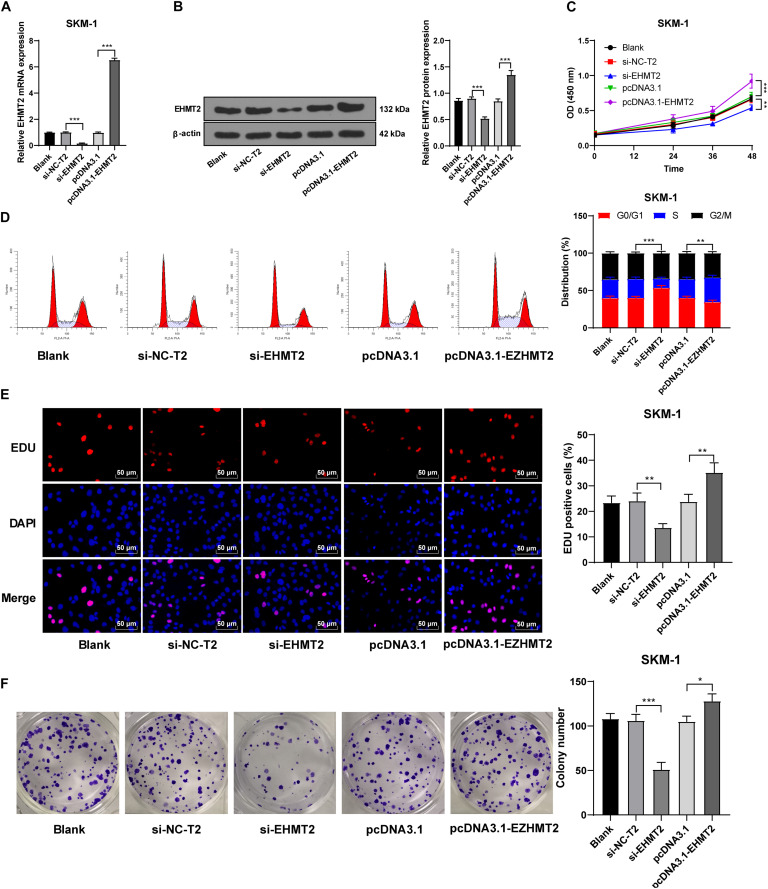
Silencing EHMT2 expression inhibited SKM-1 cell proliferation. **(A,B)** Transfection efficiency of si-EHMT2 or overexpression of EHMT2 was confirmed using RT-qPCR and Western blot; **(C)** Viability of SKM-1 cells under different treatments was measured using CCK-8 assay; **(D)** Cell cycle of SKM-1 cells was detected using flow cytometry; **(E,F)** Proliferation ability of SKM-1 cells was measured using EdU and colony formation assay. The cell experiments were repeated three times. Data are expressed as mean ± standard deviation. Data were analyzed using one-way ANOVA, followed by Tukey’s multiple comparisons test, *p < 0.05, **p < 0.01, ***p < 0.001.

SKM-1 cells were also transfected with si-EHMT2 or pcDNA3.1-EHMT2. The results of RT-qPCR and Western blot confirmed the transfection efficiency (*p* < 0.05; Figures 4A,B). Compared with the untransfected cells, SKM-1 cells transfected with si-EHMT2 showed notably reduced proliferation ability and blocked cell cycle, while SKM-1 cells transfected with pcDNA3.1-EHMT2 had enhanced proliferation ability (all *p* < 0.05; Figures 4C–F). Briefly, EHMT2 promoted SKM-1 cell proliferation.

### EZH2 Regulated H3K27me3 Level and EZH1 Compensated for the Effect of EZH2 Deficiency

**FIGURE 5:**
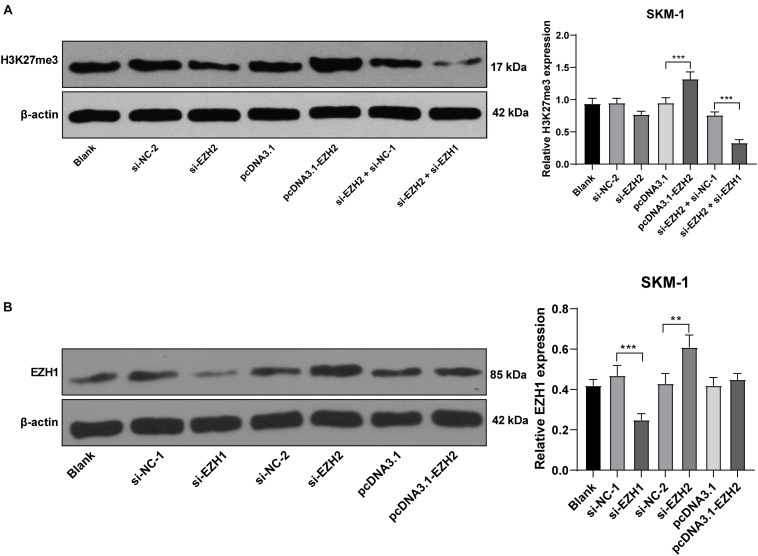
EZH2 regulated H3K27me3 level and EZH1 compensated for the effect of EZH2 deficiency. SKM-1 cells were transfected with si-EZH1, pcDNA3.1-EZH2, or si-EZH2. **(A)** H3K27me3 level in SKM-1 cells was measured using Western blot analysis; **(B)** EZH1 protein level in SKM-1 cells was detected using Western blot analysis. Image J was used for the gray analysis of the Western blot bands. The cell experiments were repeated three times. Data were analyzed using one-way ANOVA, followed by Tukey’s multiple comparisons test, **p < 0.01, ***p < 0.001.

EZH2 is an epigenetic regulator that regulates gene transcription by promoting H3K27me3 methylation level (Hosono, 2019). SKM-1 cells were transfected with pcDNA3.1-EZH2 or si-EZH2, and the transfection efficiency is shown in Figures 3A,B. Western blot revealed that H3K27me3 level was increased notably in SKM-1 cells after EZH2 overexpression, but did not decrease after EZH2 silencing (*p* > 0.05; Figure 5A). The previous literature has demonstrated that EZH1 has overlapping mechanism with EZH2 in MDS (Ling et al., 2019). EZH1 expression was increased significantly when EZH2 was knocked down (*p* < 0.05; Figure 5B). Therefore, we speculated that the reason why H3K27me3 did not decrease when EZH2 was knocked down may be due to the functional compensation of EZH1. Subsequently, we intervened the expressions of EZH1 and EZH2 in SKM-1 cells simultaneously (Figure 5B shows the transfection efficiency of si-EZH1), and found that H3K27me3 level in SKM-1 cells was notably decreased (*p* < 0.05; Figure 5A).

### EHMT2 Positively Regulated H3K9me1/H3K9me2 Level

**FIGURE 6:**
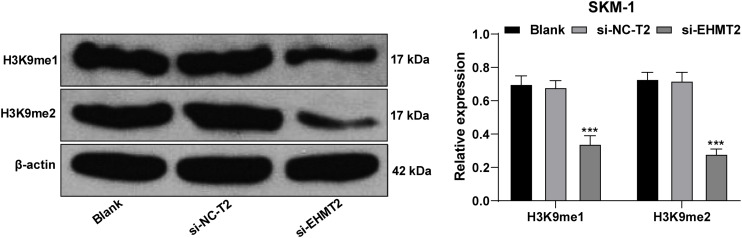
EHMT2 positively regulated H3K9me1/H3K9me2 level. Protein levels of H3K9me1/H3K9me2 in SKM-1 cells were measured using Western blot analysis. The cell experiments were repeated three times. Data were analyzed using one-way ANOVA, followed by Tukey’s multiple comparisons test, ***p < 0.001 vs. Blank group.

Similar to EZH2, EHMT2 inhibits the transcription of tumor suppressor genes by promoting the methylation and dimethylation of H3K9me1/3K9me2 (Kondengaden et al., 2016). We detected the levels of H3K9me1 and H3K9me2 in SKM-1 cells. H3K9me1 and H3K9me2 were decreased significantly in SKM-1 cells transfected with si-EHMT2 (*p* < 0.001) (Figure 6).

### EZH2 and EHMT2 Synergistically Inhibited DLX5 Gene Transcription

**FIGURE 7:**
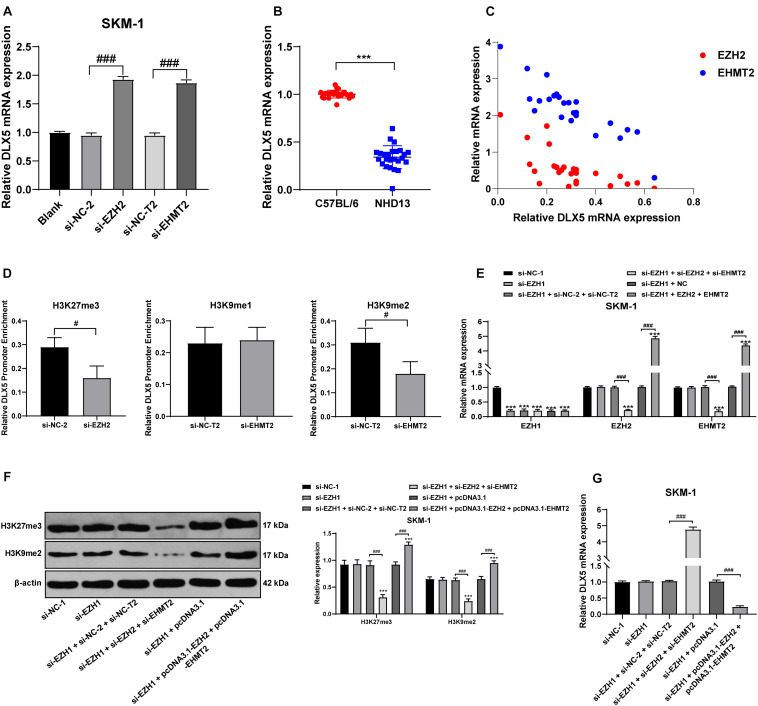
EZH2 and EHMT2 synergistically inhibited DLX5 gene transcription. **(A)** DLX5 mRNA expression in SKM-1 cells was detected using RT-qPCR; **(B)** DLX5 mRNA expression in NHD13 mice and C57BL/6 mice was detected using RT-qPCR; **(C)** DLX5 mRNA expression was increased to the highest level when EZH2 and EHMT2 were at low expression at the same time, and decreased to the lowest level when EZH2 and EHMT2 were at high expression at the same time, with the *X*-axis representing DLX5 mRNA expression, the *Y*-axis representing EZH2 and EHMT2 mRNA expressions; **(D)** Binding levels of DLX5 promoter region with H3K27me3, H3K9me1, and H3K9me2 were detected using ChIP, with the histogram showing the binding content of DLX5 promoter in DNA IP detected using RT-qPCR after ChIP experiment, in the presence of the relative content of control DNA input; **(E)** Expressions of EZH1, EZH2, and EHMT2 in SKM-1 cells were detected using RT-qPCR; **(F)** Expressions of H3K27me3 and H3K9me2 in SKM-1 cells were detected using Western blot analysis; **(G)** DLX5 mRNA expression in SKM-1 cells was detected using RT-qPCR. The cell experiments were repeated three times. Data were analyzed using one-way ANOVA, followed by Tukey’s multiple comparisons test, ***p < 0.001 vs. si-NC-1 group; ^#^*p* < 0.05 vs. adjacent groups, ^###^*p* < 0.001 vs. adjacent groups.

The high methylation and low expression of DLX5 are frequent in AML and MDS, and are related to the transformation from MDS to AML (Zhang et al., 2020). We detected the transcription level of DLX5 in SKM-1 cells of each group and found that transfection of si-EZH2 alone or si-EHMT2 alone could promote the DLX5 mRNA level (Figure 7A). DLX5 mRNA level in NHD13 mice was lower than that in healthy C57BL/6 mice (Figure 7B). DLX5 mRNA level was increased to the highest level when EZH2 and EHMT2 were at low expressions at the same time, and decreased to the lowest level when EZH2 and EHMT2 were at high expressions (Figure 7C) (all *p* < 0.001). Therein, we speculated that the transcription of DLX5 was co-inhibited by EZH2 and EHMT2.

To confirm the specific mechanism of EZH2 and EHMT2 regulating DLX5 transcription, we detected the binding levels of DLX5 promoter region with H3K27me3, H3K9me1, and H3K9me2 in SKM-1 cells. The binding level of H3K27me3 and H3K9me2 to DLX5 promoter was significantly decreased in SKM-1 cells transfected with si-EZH2 or si-EHMT2, while the binding rate of H3K9me1 to DLX5 promoter was not affected by si-EHMT2 (*p* < 0.05) (Figure 7D). These results indicated that EZH2 and EHMT2 synergistically catalyze H3K27me3 and H3K9me2 to inhibit DLX5 transcription in SKM-1 cells.

Then EZH1-treated SKM-1 cells were simultaneously transfected with overexpressing EZH2 and EHMT2 or silencing EZH2 and EHMT2 (Figure 7E), which made H3K27me3 and H3K9me2 increase or decrease simultaneously (Figure 7F). The mRNA level of DLX5 was decreased in the cells overexpressing EZH2 and EHMT2, but increased in the cells silencing EZH2 and EHMT2 (all *p* < 0.001) (Figure 7G). Taken together, EZH2 and EHMT2 synergistically inhibited DLX5 gene transcription in SKM-1 cells.

### Histone Demethylation Enhanced DLX5 Expression and Inhibited SKM-1 Cell Proliferation

**FIGURE 8:**
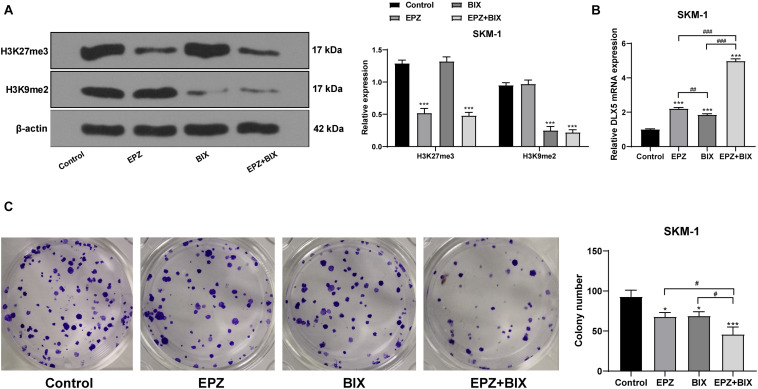
Histone demethylation enhanced DLX5 expression and inhibited SKM-1 cell proliferation. SKM-1 cells transfected with si-EZH1, pcDNA3.1-EZH2, and pcDNA3.1-EHMT2 were used as the control group, and then treated with EPZ-6438 and (or) BIX-01294. **(A)** Histone methylation and protein level were measured using Western blot analysis; **(B)** DLX5 mRNA expression was detected using RT-qPCR; **(C)** Proliferation ability of SKM-1 cells under different treatments was measured using colony formation assay. The cell experiments were repeated three times. Data were analyzed using one-way ANOVA, followed by Tukey’s multiple comparisons test, *p < 0.05, vs. Control group, ***p < 0.001 vs. Control group; ^#^*p* < 0.05 vs. adjacent groups, ^##^*p* < 0.01 vs. adjacent groups, ^###^*p* < 0.001 vs. adjacent groups.

To verify that the transcription of DLX5 was co-inhibited by H3K27me3 and H3K9me2, we used SKM-1 cells transfected with si-EZH1 and overexpressing EZH2 and EHMT2 as the control group, and treated the cells with 5 μM EZH2 inhibitor EPZ-6438 for 4 days and/or 2.5 μM EHMT2 inhibitor BIX-01294 for 3 days, respectively. It was found that DLX5 mRNA expression was increased whether H3K27me3 or H3K9me2 was inhibited alone, but the increase of DLX5 mRNA expression was the highest when H3K27me3 and H3K9me2 were inhibited at the same time (Figures 8A,B). To avoid the off-target effect of the inhibitors, we also used the EZH2 inhibitor PF-0672630 or EHMT2 inhibitor BRD4770 to treat SKM-1 cells, and compared the levels of H3K27me3 or H3K9me2. The results showed that there was no difference in the effect between inhibitors at the same target (Supplementary Figure 1). The increase of DLX5 expression inhibited the proliferation of SKM-1 cells (Figure 8C). All in all, EZH2 and EHMT2 synergistically inhibited the transcription of DLX5 in MDS cells and then promoted the transformation from MDS to AML.

## Discussion

Myelodysplastic syndrome has a high tendency to developing into AML and shows a poor outcome especially in the relapsed and older patients (Maegawa et al., 2014; Haroun et al., 2017). Epigenetic dysregulation has been considered to be associated with the pathogenesis of MDS/AML (Hosono, 2019). Histone methyltransferase is an epigenetic regulator, and its potential therapeutic effect in MDS as a small molecule inhibitor has aroused great interests (Xu and Li, 2012). This study elucidated that histone methyltransferases EZH2/EHMT2 exerted synergistic promoting effects on the transformation of MDS to AML.

EZH2 as a histone methyltransferase, exerts effects on the equilibrium between self-renewal and differentiation of hematopoietic stem cells (Safaei et al., 2018) and represents an independent prognostic factor of MDS (Nazha et al., 2016). EHMT2 can interact with transcription factors and participate in the regulation of MDS and AML (Spensberger and Delwel, 2008). We showed that EZH2 expression was reduced in bone marrow of MDS patients and promoted in MDS-AML patients, and EHMT2 was always highly expressed in MDS and MDS-AML patients. Additionally, NHD13 mice showed decreased EZH2 expression and increased EHMT2 expression. NHD13 mice with high expression of EZH2 or EHMT2 rapidly transformed from MDS to AML in a short time, and the survival rate was also notably reduced. He et al. (2019) have also clarified that EZH2 is associated with drug resistance and deterioration of MDS, as well as the progression from MDS to AML. Lehnertz et al. (2014) have shown that EHMT2 inhibitor can significantly delay the progression of disease and reduce the frequency of leukemia stem cells in a mouse model of AML. These results suggested that the expressions of EZH2/EHMT2 were concerned with the transformation from MDS to AML.

There is an interplay between EZH2 and EHMT2 to jointly maintain the silence of developmental gene subset (Mozzetta et al., 2014). The recent publication provides a strong theoretical basis to demonstrate that the dual inhibition of EZH2/EHMT2 methyltransferases can bring more effective prospects for cancer treatment (Soumyanarayanan and Dymock, 2016). Consistently, we showed that silencing EZH2/EHMT2 expression significantly reduced SKM-1 cell proliferation and blocked cell cycle.

EZH2 establishes H3K27me3 markers on specific genes to promote the transcriptional inhibition of target genes (Safaei et al., 2018). H3K27me3 level was increased notably when EZH2 was overexpressed, but it did not decrease significantly when EZH2 was knocked down. The effect of EZH2 deficiency may be limited due to EZH1 compensation and overlapping mechanism of transformation (Ling et al., 2019). The biological significance of EZH1 is viewed as a backup enzyme of EZH2, which can make up for EZH2 deficiency in transcriptional inhibition in hematopoietic cells (Margueron et al., 2008; Ezhkova et al., 2011; Mochizuki-Kashio et al., 2015). EZH1 expression in SKM-1 cells was increased significantly in the absence of EZH2. Downregulation of EZH2 reduced the level of H3K27me3 in SKM-1 cells after intervention of EZH1 expression. EZH1 targets bivalent genes to sustain the self-renewal of stem cells in EZH2-deficient MDS (Aoyama et al., 2018). Briefly, EZH2 regulated the level of H3K27me3, and EZH1 compensated for the effect of EZH2 deletion. Similar to EZH2, EHMT2 inhibits the transcription of tumor suppressors by promoting the levels of H3K9me1/H3K9me2 (Kondengaden et al., 2016). H3K9me1/H3K9me2 level in SKM-1 cells were reduced significantly after intervention of EHMT2. EZH2/EHMT2 exert effects on SKM-1 cell proliferation by modulating the H3K27me3 and H3K9me1/H3K9me2 level.

Curry et al. (2015) have revealed that EZH2/EHMT2 dual inhibition induces gene transcription and inhibits cancer cell growth. The DLX genes serve as DNA-binding transcriptional regulators, controlling considerable downstream effector genes (Kraus and Lufkin, 2006). Notably, DLX5 hypermethylation is reported to be a common event in AML and MDS, and also concerned with the transformation from MDS to leukemia (Zhang et al., 2020). We showed that dual inhibition of EZH2 and EHMT2 in SKM-1 cells elevated the DLX5 mRNA expression to the highest level. The increase of DLX5 expression significantly inhibited the proliferation of SKM-1 cells. DLX5 expression is upregulated by methyltransferase inhibitors during odontogenic differentiation of human dental pulp cells (Zhang et al., 2015). And upregulation of DLX5 could repress SKM-1 cell proliferation. Zhang et al. (2020) have also exhibited that DLX5 has antiproliferative and pro-apoptotic influences on SKM-1 cells. Briefly, EZH2/EHMT2 synergistically catalyzed H3K27me3/H3K9me2 to inhibit the transcription of DLX5 and promoted the transformation from MDS to AML.

**FIGURE 9:**
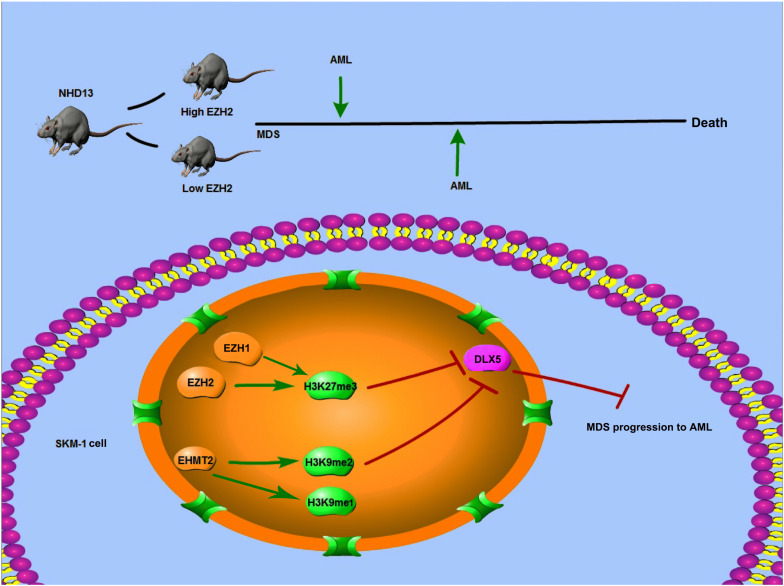
Mechanism diagram. EZH2 and EHMT2 synergistically regulated DLX5 transcription and promoted the transformation from MDS to AML. NHD13 mice with high EZH2 expression in peripheral blood had earlier AML transformation. EZH1 and EZH2 regulated the level of histone H3K27me3, and EHMT2 catalyzed H3K9me1 and H3K9me2. EZH2 and EHMT2 regulated the histone methylation level of DLX5 promoter region, synergistically inhibited the transcription of DLX5, and then promote the transformation of MDS into AML.

To sum up, EZH2/EHMT2 catalyzed H3K27me3/H3K9me2 to inhibit the transcription of DLX5, thus promoting the transformation from MDS to AML. This pilot study may provide theoretical holds for the EZH2/EHMT2-based regimens of MDS patients (Figure 9). However, there are some limitations of this study. The sample size of this study is small. To study the clinical significance of EZH2 and EHMT2, such as the relationship with clinical manifestations, genetic characteristics, and prognosis, we need more cases to support data analysis. We will collect more cases for analysis in the future study. Also, we shall carry out more prospective trials on the feasibility and safety of EZH2/EHMT2 inhibitor in the treatment of MDS, so as to refine our clinical guidance.

## Data Availability Statement

The original contributions presented in the study are included in the article/Supplementary Material, further inquiries can be directed to the corresponding author.

## Ethics Statement

The studies involving human participants were reviewed and approved by the Clinical Ethical Committee of the Second Hospital of Shanxi Medical University. The patients/participants provided their written informed consent to participate in this study. The animal study was reviewed and approved by the Clinical Ethical Committee of the Second Hospital of Shanxi Medical University.

## Author Contributions

ZZ was the guarantor of integrity of the entire study, definition of intellectual content, contributed to the literature research, and manuscript editing. YM contributed to the study concepts and manuscript review. WC contributed to the study design, data analysis, and statistical analysis. LL contributed to the manuscript preparation and clinical studies. GL, YZ, and FR contributed to the experimental studies. CD data acquisition. All authors read and approved the final manuscript.

## Conflict of Interest

The authors declare that the research was conducted in the absence of any commercial or financial relationships that could be construed as a potential conflict of interest.

## Publisher’s Note

All claims expressed in this article are solely those of the authors and do not necessarily represent those of their affiliated organizations, or those of the publisher, the editors and the reviewers. Any product that may be evaluated in this article, or claim that may be made by its manufacturer, is not guaranteed or endorsed by the publisher.

## Supplementary Material

Supplementary Figure 1 Inhibitory effect of EZH2 inhibitor and EHMT2 inhibitor on histone methylation. SKM-1 cells co-transfected with si-EZH1, pcDNA3.1-EZH2, and pcDNA3.1-EHMT2 were used as the control group. Cells in logarithmic growth phase were seeded into the 96-well plates (1 × 10^5^ cells/mL) supplemented with culture medium, and then treated with 15 μM PF-0672630 for 5 days (PF), 5 μM EPZ-6438 for 4 days (EPZ), 5 μM BRD4770 for 2 days (BRD), and 2.5 μM BIX-01294 for 3 days (BIX), respectively. The histone methylation level was detected using Western blot, ^∗^*p* < 0.05, vs. Control group, ns: *p* > 0.05.
