# Supplementary figures and images for: EZH2/EHMT2 Histone Methyltransferases Inhibit the Transcription of DLX5 and Promote the Transformation of Myelodysplastic Syndrome to Acute Myeloid Leukemia
Source: Front Cell Dev Biol. 2021 Aug 2;9:619795. doi: 10.3389/fcell.2021.619795 (PMC8365305; doi:10.3389/fcell.2021.619795)

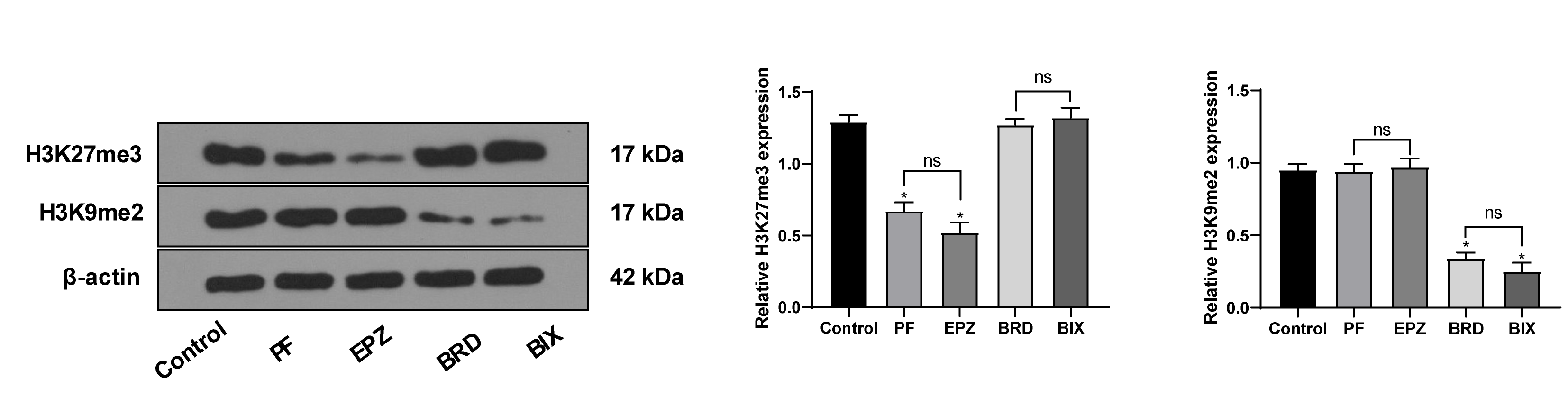

Supplement: Supplementary Figure 1 — Inhibitory effect of EZH2 inhibitor and EHMT2 inhibitor on histone methylation. SKM-1 cells co-transfected with si-EZH1, pcDNA3.1-EZH2, and pcDNA3.1-EHMT2 were used as the control group. Cells in logarithmic growth phase were seeded into the 96-well plates (1 × 10^5^ cells/mL) supplemented with culture medium, and then treated with 15 μM PF-0672630 for 5 days (PF), 5 μM EPZ-6438 for 4 days (EPZ), 5 μM BRD4770 for 2 days (BRD), and 2.5 μM BIX-01294 for 3 days (BIX), respectively. The histone methylation level was detected using Western blot, ∗p < 0.05, vs. Control group, ns: p > 0.05. [file Image_1.TIFF]
