# Supplementary material for: EZH2/EHMT2 Histone Methyltransferases Inhibit the Transcription of DLX5 and Promote the Transformation of Myelodysplastic Syndrome to Acute Myeloid Leukemia
Source: Front Cell Dev Biol. 2021 Aug 2;9:619795. doi: 10.3389/fcell.2021.619795 (PMC8365305; doi:10.3389/fcell.2021.619795)
Supplement: Supplementary file 2 [file Table_1.DOCX]

**Supplementary Table 1** Sequences for siRNAs

| siRNAs | Sequences |
| --- | --- |
| si-EZH1 | F: 5′-GCGACUUCGACAACUUAAATT-3′ |
|  | R: 5′-UUUAAGUUGUCGAAGUCGCTT-3′ |
| si-NC-1 | F: 5′-UUCUCCGAACGUGUCACGUTT-3′ |
|  | R: 5′-ACGUGACACGUUCGGAGAATT-3′ |
| si-EZH2 | F: 5′-GAGGGAAAGUGUAUGAUAATT-3′ |
|  | R: 5′-UUAUCAUACACUUUCCCUCTT-3′ |
| si-NC-2 | F: 5′-UUCUCCGAACGUGUCACGUUU-3′ |
|  | R: 5′-ACGUGACACGUUCGGAGAAUU-3′ |
| si-EHMT2 | F: 5′-CGCACAGAGAAGAUCAUCUTT-3′ |
|  | R: 5′-AGAUGAUCUUCUCUGUGCGTT-3′ |
| si-NC-T2 | F: 5′-TTCTCCGAACGTGTCACGTTT-3′ |
|  | R: 5′-AAACGTGACACGTTCGGAGAA-3′ |
